# Post-Effects of Time-Restricted Feeding against Adipose Tissue Inflammation and Insulin Resistance in Obese Mice

**DOI:** 10.3390/nu15112617

**Published:** 2023-06-02

**Authors:** Narae Yun, Jiyeon Nah, Mi Nam Lee, Dayong Wu, Munkyong Pae

**Affiliations:** 1 Department of Food and Nutrition, Chungbuk National University, Chungdae-ro 1, Seowon-gu, Cheongju 28644, Republic of Korea; skfo1999@naver.com (N.Y.); nnjj0000@naver.com (J.N.); 2 Department of Biological Sciences, Chonnam National University, 77 Yongbong-ro, Buk-gu, Gwangju 61186, Republic of Korea; nam6658@gmail.com; 3 Nutritional Immunology Laboratory, Jean Mayer USDA Human Nutrition Research Center on Aging, Tufts University, 711 Washington Street, Boston, MA 02111, USA; dayong.wu@tufts.edu

**Keywords:** after-effects, inflammation, obesity, post-effects, time-restricted feeding

## Abstract

Time-restricted feeding (TRF) has been shown to improve the disordered metabolic and immunologic functions associated with obesity, however little is known about its post-effects after the cessation of TRF practice. In the current study, we determined how long the effects of TRF persist, and whether the effects are tissue-dependent. There were four groups of mice in this study: overweight and obese mice were randomized into (1) TRF group (TRF for 6 weeks), (2) post-TRF group (TRF for 4 weeks and later ad libitum), (3) continuous ad libitum of high-fat diet (HFD-AL), and (4) the lean control-fed low-fat diet ad libitum. Blood, liver, and adipose tissues were collected to measure the metabolic, inflammatory, and immune cell parameters. The results showed that TRF withdrawal quickly led to increased body weight/adiposity and reversed fasting blood glucose. However, fasting insulin and insulin resistance index HOMA-IR remained lower in the post-TRF than in the HFD-AL group. In addition, TRF-induced reduction in blood monocytes waned in the post-TRF group, but the TRF effects on mRNA levels of proinflammatory immune cells (macrophages *Adgre1* and *Itgax*) and cytokine (*Tnf*) in adipose tissue remained lower in the post-TRF group than in the HFD-AL group. Furthermore, the TRF group was protected from the down-regulation of *Pparg* mRNA expression in adipose tissue, which was also observed in the post-TRF group to a lesser extent. The post-TRF animals displayed liver mass similar to those in the TRF group, but the TRF effects on the mRNA of inflammation markers in the liver vanished completely. Together, these results indicate that, although the lasting effects of TRF may differ by tissues and genes, the impact of TRF on adipose tissue inflammation and immune cell infiltration could last a couple of weeks, which may, in part, contribute to the maintenance of insulin sensitivity even after the cessation of TRF.

## 1. Introduction

Time-restricted feeding (TRF) is a dietary regimen with a daily eating window, usually 10 h or less, followed by at least 14 h of fasting [1,2]. Over the past few years, increasing numbers of studies have documented that rodents on TRF are largely protected from developing obesity and several metabolic diseases [3,4,5], and that those with pre-existing obesity also benefit from the therapeutic effects of TRF [6,7,8]. It appears that TRF exerts various effects on multiple tissues, including adipose tissue, bone marrow, and the liver during obesity. In mice receiving a high-fat diet, those on TRF had less severe adipose tissue inflammation compared to their counterparts fed ad libitum [6,8]. In particular, the infiltration of macrophages and expression of inflammation genes restricted to or enriched in macrophages, such as *Adgre1* (F4/80) and *Tnf* [9], were reduced in the adipose tissue of TRF mice [8]. In parallel, TRF intervention was effective in normalizing the obesity-induced expansion of myeloid progenitor populations in the bone marrow and thus the monocyte pool in circulation [10]. Mice on TRF experience extended daily fasting, which alters various hepatic gene expressions, including peroxisome proliferator-activated receptor (PPAR) [4]. While PPARγ level was elevated in the liver of obese mice [4,11], TRF effectively reduced PPARγ and the associated lipogenic gene expression, along with a reduction in fatty liver disease [4]. Additional gene expressions associated with hepatic inflammation were also either reversed or attenuated under TRF intervention [4,12,13]. 

Most of these benefits have been observed under continuous TRF conditions, and, whilst this is not clear, it is important to know whether these beneficial effects persist after cessation of TRF. This is important before any human trial begins, because individuals often encounter difficulties due to family, social, or work life [14], and may stop TRF intervention. Previously, Chaix et al. reported that mice under 5 days of TRF and 2 days of ad libitum had hepatic gene expression signatures similar to continuous TRF and exhibited improved metabolic parameters compared to those under ad libitum [6]. Although these results suggest that temporal interruption during the weekend may not deter the health benefits of TRF, it remains to be determined whether this can last beyond two days. 

The present study aimed to investigate the post-effects of TRF after 2 weeks of cessation on body weight, insulin resistance index, inflammatory responses in adipose tissue and the liver, and on the circulating immune cells of obese mice. By comparing with continuous TRF or ad libitum, this could provide insights into whether 2 weeks of TRF withdrawal could maintain its effect—and thus be worth trying even with a risk of failure—or could further worsen insulin resistance and metabolic tissue inflammation, which may deter individuals from TRF cessation.

## 2. Materials and Methods

### 2.1. Animals and Diets

All animal experiments were reviewed and approved by the Institutional Animal Care and Use Committee of Chungbuk National University (approval number: CBNUA-1602-21-01). Five-week-old male C57BL/6J mice were purchased from the Central Laboratory Animal Inc. (Seoul, Republic of Korea) and housed 3–4 mice per cage under controlled temperature (23 °C ± 1 °C), relative humidity (50% ± 10%), and light/dark cycle (12-h dark/12-h light 7:00 a.m.–7:00 p.m.). After 1 week of adaptation, mice were randomly assigned into 2 groups and fed a low-fat diet (LFD; 10% calories from fat, D12450B; Research Diets, New Brunswick, NJ, USA) or a high–fat diet (HFD; 60% calories from fat, D12492; Research Diets) ad libitum. At 6 weeks, when the HFD-fed mice weighed around 40 g, the HFD-fed mice were randomly divided into 3 subgroups: (1) ad libitum feeding (HFD-AL), (2) TRF for 6 weeks (TRF), or (3) TRF for 4 weeks, followed by 2 weeks of ad libitum feeding (post-TRF) (16–18 mice/group). TRF animals had access to HFD for 10 h between ZT13 (1 h after lights off) and ZT23 (1 h before lights on). Food access was controlled by transferring mice daily between cages with food and water, and cages with water only. To control the mouse handling, ad-libitum-fed mice were also transferred between feeding cages at the same time. Food intake was measured twice a week by monitoring the weight of the remaining food. Body weight was measured once a week. After 12 weeks on the feeding regimen, blood was collected from tail veins of unanesthetized mice after a 6 h fast (ZT23-ZT5) to measure whole blood glucose and circulating leukocytes. Immediately after euthanasia, blood was collected by cardiac puncture and tissues were harvested at 1 p.m. (ZT6).

### 2.2. Measurements of Blood Glucose, Serum Insulin, and HOMA-IR

Whole blood glucose levels were measured from the tail veins using a Contour Plus glucometer (Ascensia Diabetes Care, Basel, Switzerland). Blood serum was isolated using BD Microtainer tube with serum separator (365967, Becton Dickinson, Franklin Lakes, NJ, USA) according to the manufacturer’s instructions. Serum insulin was determined by Ultra Sensitive Mouse Insulin ELISA kit (90080, Crystal Chem, Downers Grove, IL, USA). Homeostatis model assessment of insulin resistance (HOMA-IR) was calculated as fasting blood glucose (mmol/L) × fasting insulin (mU/L)/22.5 [15].

### 2.3. Histologic Analysis

Left epididymal pads and liver tissues were fixed in 4% formaldehyde (Sigma, St. Louis, MO, USA) overnight, embedded in paraffin, sectioned, and stained with hematoxylin and eosin (H&E). Additionally, liver specimens were stained with Oil-red-O to illustrate hepatic lipid accumulation. Digital images were acquired with a Lionheart FX automated microscope (BioTek, Winooski, VT, USA). The size of the adipocyte area was determined using Image J software (National Institutes of Health, Bethesda, MD, USA).

### 2.4. RNA Extraction and Quantitative Real-Time PCR 

Right epididymal pads and liver tissues were immediately frozen in liquid nitrogen and stored at −70 °C for gene expression analysis. RNA was isolated from pulverized adipose tissue and liver using an RNeasy Lipid Mini Kit (74804, Qiagen, Valencia, CA, USA) according to the manufacturer’s instructions. One microgram of total RNA was converted to double-stranded cDNA with an Advantage RT for PCR kit (639506, Clontech, Palo Alto, CA, USA). Quantitative PCR was performed on a QuantStudio 5 Real-Time PCR system (Applied Biosystems, Foster City, CA, USA) using TaqMan Universal PCR Master Mix (4304437, Applied Biosystems) and primers for *Adgre1* (Mm00802529_m1), *Adipoq* (Mm00456425_m1), *Ccl2* (Mm00441242_m1), *Ccl8* (Mm01297183_m1), *Cd3* (Mm01179194_m1), *Cd4* (Mm00442754_m1), *Cd8* (Mm01182107_g1), *Elane* (Mm00469310_m1), *Il6* (Mm00446190_m1), *Itgax* (Mm00498698_m1), *Ppara* (Mm00440939_m1), *Pparg* (Mm00440940_m1), *Tbp* (Mm00446973_m1), and *Tnf* (Mm00443258_m1) (all FAM probes, Applied Biosystems). Fold changes were calculated as 2^−ΔΔCt^ compared with the endogenous control gene, TATA box-binding protein (TBP), using LFD-AL as the reference group.

### 2.5. Flow Cytometric Analysis of Circulating Leukocytes

Blood was collected from tail veins in the presence of 5 mM EDTA, incubated with BD Fc Block for 10 min, and then stained with mAbs (BD Bioscience unless indicated otherwise) specific to CD45-APC-Cy7 (clone 30-F11), Ly6G-eFluor450 (1A8-Ly6g, eBioscience), CD11b-PerCP-Cy5.5 (M1/70), Ly6C-FITC (AL-21), NK1.1-APC (PK136), CD3-APC (145-2C11), CD19-APC (1D3), and TER119-APC (TER-119, eBioscience) for 20 min at room temperature. The samples were then fixed and lysed using BD FACS Lysing solution (BD Bioscience). After fixation, cells were washed and resuspended in 2% FBS/PBS. AccuCheck counting beads (PCB100, Invitrogen, Grand Island, NY, USA) were used for quantification of absolute cell numbers. Multiparameter analysis was performed on a FACSSymphony A3 (BD Bioscience) using the FACSDiva software and data were analyzed using the FlowJo software (version 10, Tree Star Inc., Ashland, OR, USA) as described previously [10].

### 2.6. Statistical Analysis

Results were expressed as means ± SEM. Data were analyzed using ANOVA followed by Tukey’s HSD post hoc procedure. Pearson’s correlation test was conducted to evaluate the association between variables. All statistical analyses were carried out using SPSS statistical software version 25.0 (Chicago, IL, USA), with *p* values smaller than 0.05 considered significant.

## 3. Results

### 3.1. Cessation of TRF Did Not Rescue Body Weight Gain and Fat Deposition Associated with High-Fat Diet

**Figure 1:**
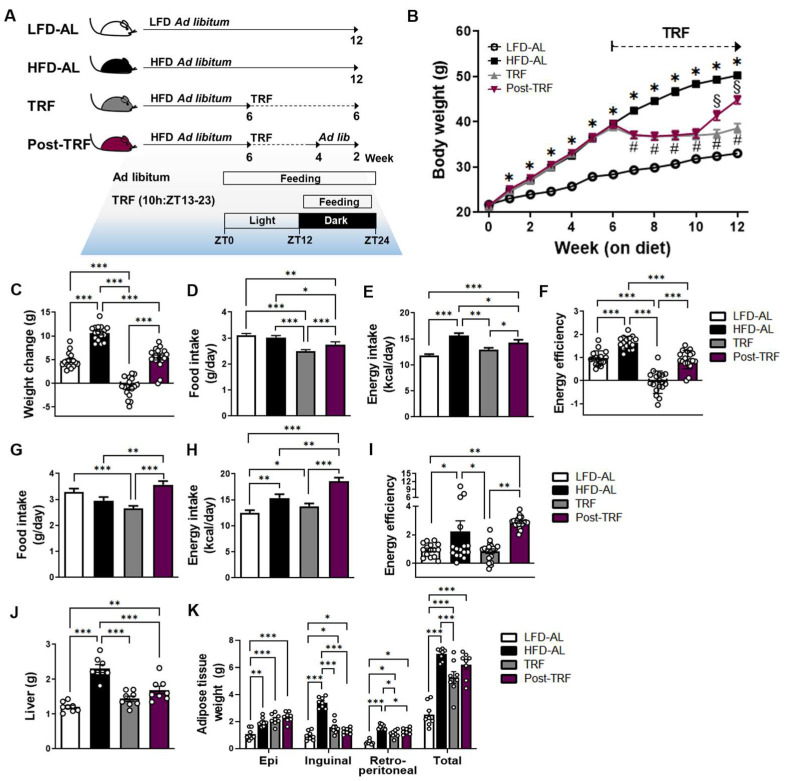
Post-effects of TRF on body weight gain, energy efficiency, and tissue weights. (**A**) Schematic outline of the four feeding regimens used in this study. Six-weeks of C57BL/6 male mice were randomly assigned into two groups and fed the low-fat diet (LFD) or 60% high-fat diet (HFD) ad libitum for 6 weeks until 12 weeks of age. The HFD-fed mice were further divided into three subgroups; ad libitum feeding (HFD-AL) or 10 h time-restricted feeding during active period (TRF) for another 6 weeks, or TRF for another 4 weeks followed by 2 weeks of ad libitum feeding (post-TRF). (**B**) Average body weight, *n* = 16–18 per group. * *p* < 0.001, # *p* < 0.001, and § *p* < 0.001, significantly different at same week on diets by ANOVA with Tukey’s post hoc test. (**C**) Body weight change, (**D**) daily food intake, (**E**) daily energy intake, and (**F**) energy efficiency (g gain/kcal consumed) during the 6 weeks of TRF period. (**G**) Daily food intake, (**H**) daily energy intake, and (**I**) energy efficiency during the last 2 weeks of the TRF cessation (ad libitum) period. (**J**) Liver weight, *n* = 8 per group. (**K**) Fat distribution: epididymal adipose tissue, inguinal adipose tissue, retroperitoneal adipose tissue, *n* = 8 per group. Data are presented as means ± SEM. * *p* < 0.05, ** *p* < 0.01, *** *p* < 0.001 by ANOVA with Tukey’s post hoc test.

To explore how long the effects of TRF persist in body weight and fat deposition, we subjected overweight and obese mice to 4 weeks of TRF followed by 2 weeks of TRF withdrawal (post-TRF group; Figure 1A). Prior to the TRF intervention, mice weighed around 40 g on average, after being fed HFD ad libitum for 6 weeks. Over the 6 weeks of TRF intervention, the mice were resistant to weight gain, even with HFD feeding (Figure 1B,C). They had similar calorie intake but lower energy efficiency (weight gain/caloric consumption), compared to those in the LFD-AL group (Figure 1D–F). At 1 week after the cessation of TRF, the mice immediately gained enough weight to be significantly different from the TRF group (Figure 1B). This is due to the increased food and calorie intake compared to the TRF group, and even more so than the HFD-AL group, during the 2 weeks of cessation period (Figure 1G–I). However, the final body weight (Figure 1B) and energy efficiency (Figure 1F) were significantly lessened in the post-TRF group in comparison to the HFD-AL group, and this is largely attributed to the prior TRF effects. 

In addition, the mice in the post-TRF group had lower liver weight than those in the HFD-AL group (Figure 1J), but the total and epididymal adipose tissue weight were comparable to the HFD-AL group (Figure 1K). These results suggest that, while the cessation of TRF did not prevent the weight gain and fat deposition associated with HFD, the beneficial effects of TRF on the final body weight and energy efficiency persisted even after 2 weeks of cessation, albeit to a lesser degree.

### 3.2. Prior TRF Conveys Short-Term Effects against Development of Adipose Tissue Inflammation

**Figure 2:**
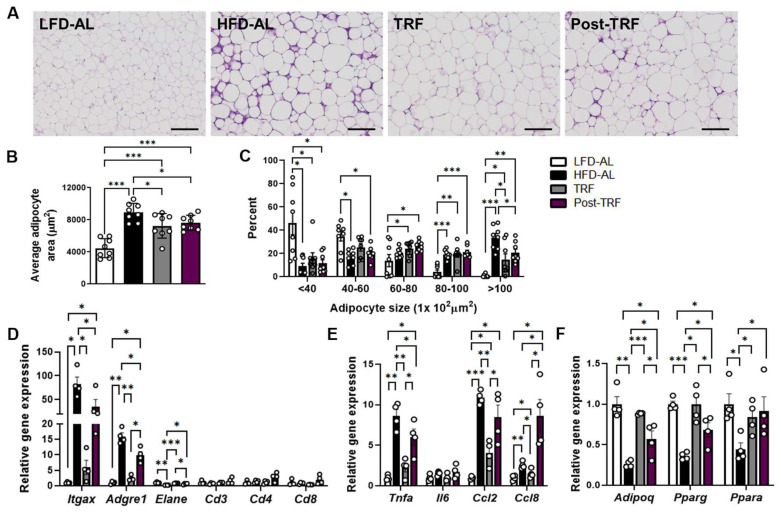
Post-effects of TRF on adiposity and adipose tissue inflammation. (**A**) Representative H&E-stained histological sections of epididymal adipose tissues. Scale bar, 200 μm. (**B**) Average adipocyte size area and (**C**) distribution of adipocytes by size category, *n* = 8 per group. (**D**) Quantitative real-time PCR analysis of proinflammatory macrophage (F4/80 and CD11c) and other immune cell markers (neutrophil elastase, CD3, CD4, CD8), (**E**) cytokines (TNFα, IL-6), chemokines (Ccl2, Ccl8), (**F**) adiponectin, and PPARs in epididymal fat, *n* = 4 per group. LFD-AL, low-fat diet ad libitum for 12 weeks; HFD-AL, high-fat diet ad libitum for 12 weeks; TRF, 6 weeks of time-restricted feeding in mice that underwent ad libitum feeding of high-fat diet for 6 weeks; Post-TRF, 4 weeks of TRF, followed by 2 weeks of ad libitum feeding in those that underwent ad libitum feeding of high-fat diet for 6 weeks. Data are presented as means ± SEM. * *p* < 0.05, ** *p* < 0.01, *** *p* < 0.001 by ANOVA with Tukey’s post hoc test.

Next, we investigated whether prior TRF contributes to attenuating adipose tissue inflammation. Despite being similar in mass (Figure 1K), the H&E-stained sections of epididymal adipose tissue in HFD-AL showed characteristic crown-like structures, a histologic hallmark of inflammation in adipose tissue, which were distinctly less severe in the post-TRF group (Figure 2A). In addition, the mice in the post-TRF group contained smaller adipocytes than those found in the HFD-AL group animals (Figure 2B,C). This phenotype coincided with lower mRNA expressions of several proinflammatory genes (Figure 2D).

We observed that the mRNA expression of the macrophage-associated gene *Adgre1* (F4/80) and the putative M1 inflammatory marker *Itgax* (CD11c) were upregulated in the adipose tissue of the HFD-AL group, which were less significant in the post-TRF group and, to a greater extent, in the TRF group (Figure 2D). There was no significant difference in the mRNA expression of the T cell markers *Cd3*, *Cd4*, and *Cd8* among the groups. In contrast, the mRNA expression of neutrophil elastase gene *Elane* was reduced in the HFD-AL group, which were fully recovered in the TRF group and, to a lesser extent, in the post-TRF group. The HFD-AL group exhibited a significant elevation of *Tnf* (TNFα) mRNA level in the adipose tissue (8.6-fold), whereas this was lower in the TRF and post-TRF groups by 73.7% and 30.5%, respectively, compared to the HFD-AL group (Figure 2E). Interestingly, the mRNA expressions of chemokine *Ccl2* and *Ccl8* in the post-TRF group were comparable to, or even greater than, the HFD-AL group (Figure 2E), suggesting that the infiltration of the immune cells into the adipose tissue may keep going on after the TRF interruption.

PPARγ is considered the master regulator of adipogenesis [16] and its activation ameliorates the insulin resistance and inflammation associated with obesity [9,17,18]. We confirmed that the *Pparg* mRNA expression was reduced in the adipose tissue of the HFD-AL mice, whereas the TRF group maintained a similar level to that in the LFD-AL group (Figure 2F). The cessation of TRF for 2 weeks led to a significant reduction in the *Pparg* mRNA expression, but its level was still approximately 1.90-fold higher than that in the HFD-AL group. PPARα is known to play a pivotal role in fatty acid catabolism and the regulation of inflammatory processes, mainly by inhibiting inflammatory gene expression [19]. While the mRNA expression of *Ppara* was downregulated in the HFD-AL group compared to the LFD-AL group, both the TRF and post-TRF groups maintained comparable levels to those in the LFD-AL group (Figure 2F). Adiponectin, an adipokine secreted by adipocytes, is a homeostatic factor for regulating lipid metabolism, insulin sensitivity, and inflammation [20]. While adiponectin expression was decreased in the adipose tissue of the HFD-AL mice, those in the TRF group, and also, to a lesser degree, in the post-TRF group, maintained their levels towards the LFD-AL (Figure 2F). These results imply that the cessation of TRF may temporarily maintain healthy visceral fat expansion despite elevated *Ccl2* and *Ccl8* expression, potentially through the preserved effect of TRF on the expression of the adipose genes linked to adipogenesis and inflammation.

### 3.3. Post-Effects of TRF on Gene Expression of Inflammatory Factors in the Liver

**Figure 3:**
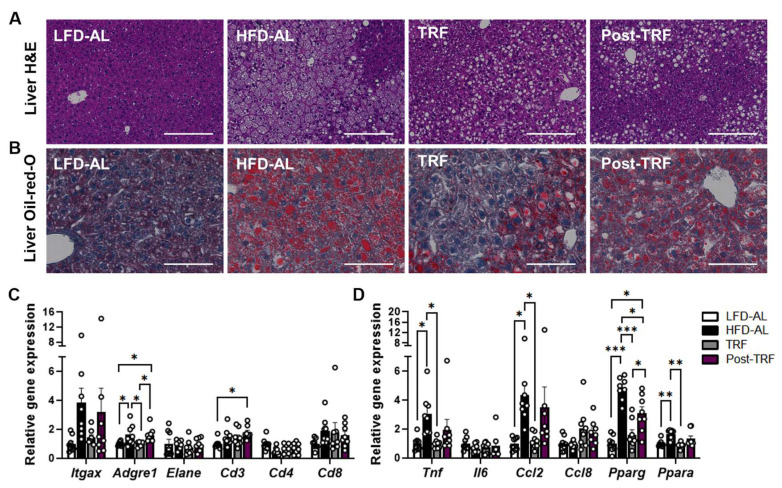
Post-effects of TRF on gene expression of inflammatory factors in the liver. (**A**) Representative images of liver sections stained with H&E. Scale bar, 200 μm. (**B**) Representative images of liver sections stained with Oil-red-O. Scale bar, 100 μm. (**C**) Quantitative real-time PCR analysis of proinflammatory macrophage (F4/80 and CD11c) and other immune cell markers (neutrophil elastase, CD3, CD4, CD8), (**D**) cytokines (TNFα, IL-6), chemokines (Ccl2, Ccl8), and PPARs in liver. LFD-AL, low-fat diet ad libitum for 12 weeks; HFD-AL, high-fat diet ad libitum for 12 weeks; TRF, 6 weeks of time-restricted feeding in mice underwent ad libitum of high-fat diet for 6 weeks; Post-TRF, 4 weeks of TRF, followed by 2 weeks of ad libitum feeding in those underwent ad libitum of high-fat diet for 6 weeks. Data are presented as means ± SEM, *n* = 8 per group. * *p* < 0.05, ** *p* < 0.01, *** *p* < 0.001 by ANOVA with Tukey’s post hoc test.

Histological examination with both H&E and Oil-red-O staining of liver sections indicated that TRF animals were protected from lipid accumulation and hepatic morphological abnormality in size and shape, which are frequently found in obese liver (Figure 3A,B). This phenotype coincided with lower liver mRNA expressions of several pro-inflammatory genes (Figure 3C,D). While the levels of mRNA encoding inflammatory factors *Adgre1* (F4/80), *Tnf* (TNFα) as well as *Ccl2* (Mcp1) were significantly higher in the liver of HFD-AL group than those of LFD-AL group, continuous TRF intervention even with HFD consumption was effective in reducing their levels similar to what seen in LFD-AL group. However, at 2 weeks after TRF cessation, mice exhibited mRNA expressions of inflammatory and chemokine factors similar to the levels in HFD-AL groups. In contrary, changes in hepatic expression of *Pparg*, a characteristic of obese subjects [21], were prevented by TRF intervention [4], and it was still partially reduced in post-TRF group so that its levels were between those seen in TRF and HFD-AL groups (Figure 3D). Similarly, higher expression of *Ppara* in the liver of obese mice [11] was reduced in TRF group, which was not sustained in post-TRF group (Figure 3D). These data suggest that at 2 weeks after cessation, the inhibitory effect of TRF on liver inflammatory markers vanished while its suppression on mRNA expression of *Pparg* was partially maintained.

### 3.4. Post-Effects of TRF on Circulating Monocyte Pool in Association with Adipose Tissue Inflammation

**Figure 4:**
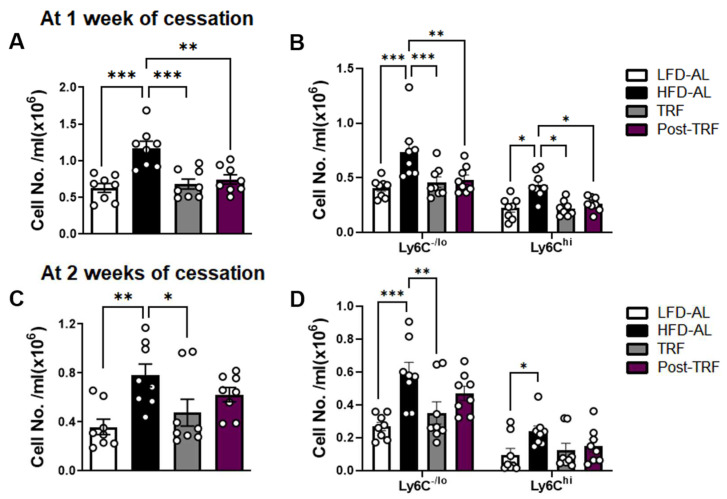
Post-effects of TRF on circulating monocyte pool in association with adipose tissue inflammation markers. (**A**) Absolute numbers of monocyte (CD45^+^NK1.1^−^CD19^−^TER119^−^CD11b^+^) and (**B**) monocyte subpopulations based on Ly6C expression levels were measured in the blood of mice after 1 week of TRF interruption. (**C**) Absolute numbers of monocyte and (**D**) monocyte subpopulation after 2 weeks of TRF interruption. LFD-AL, low-fat diet ad libitum for 11–12 weeks; HFD-AL, high-fat diet ad libitum for 11–12 weeks; TRF, 5–6 weeks of time-restricted feeding in mice underwent ad libitum of high-fat diet for 6 weeks; Post-TRF, 4 weeks of TRF, followed by 1 or 2 weeks of ad libitum feeding in those underwent ad libitum of high-fat diet for 6 weeks. Data are presented as means ± SEM, *n* = 8 per group. * *p* < 0.05, ** *p* < 0.01, *** *p* < 0.001 by ANOVA with Tukey’s post hoc test.

Previously, we demonstrated that TRF intervention effectively normalized circulating monocytes by controlling monocyte progenitor cells in the bone marrow of obese mice [8]. Thus, we analyzed the post-TRF effect on circulating monocytes by flow cytometry. At 1 week after the cessation of TRF, the mice had reduced numbers of total and Ly6C^hi^ proinflammatory monocytes, similar to the levels of those in the TRF group, as well as in the LFD-AL group (Figure 4A,B). However, these changes were not maintained at 2 weeks following TRF withdrawal (Figure 4C,D).

We next investigated whether circulating monocytes are associated with gene expression in adipose tissue. We found a positive association of circulating monocytes with the gene expression of *Adgre1* (*r* = 0.899, *p* < 0.001, Supplementary Figure S1A) and putative M1 inflammatory marker *Itgax* (*r* = 0.676, *p* < 0.01, Supplementary Figure S1B) in adipose tissue. Similarly, Ly6C^hi^ monocytes were associated with *Adgre1* (*r* = 0.710, *p* < 0.01) and *Itgax* (*r* = 0.512, *p* < 0.05) gene expression.

### 3.5. Post-Effects of TRF on Fasting Blood Glucose, Fasting Insulin, and HOMA-IR

**Figure 5:**
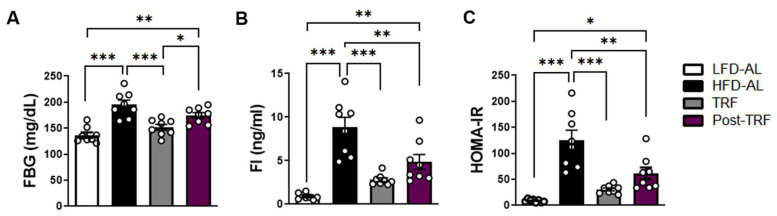
Post-effects of TRF on fasting blood glucose, fasting insulin, and insulin resistance index (HOMA-IR). Six weeks of C57BL/6 male mice were randomly assigned into two groups and fed a low-fat diet (LFD) or 60% high-fat diet (HFD) ad libitum for 6 weeks until 12 weeks of age. The HFD-fed mice were further divided into three subgroups: ad libitum feeding (HFD-AL); 10 h time-restricted feeding during active period (TRF) for another 6 weeks; or TRF for another 4 weeks followed by 2 weeks of ad libitum feeding (post-TRF). (**A**) Fasting blood glucose (FBG), (**B**) FI (fasting insulin), and (**C**) insulin resistance index (HOMA-IR), measured as FBG (mmol/L) x FI (mU/L)/22.5 [15]. Data are presented as means ± SEM, *n* = 8 per group. * *p* < 0.05, ** *p* < 0.01, *** *p* < 0.001 by ANOVA with Tukey’s post hoc test.

Given our finding that the post-TRF group sustained a partial effect on circulating monocytes and adipose tissue inflammation, we wished to learn whether the post-TRF group conveys protection against fasting blood glucose (FBG), fasting insulin (FI), and insulin resistance index HOMA-IR. We found that, while HFD increased the FBG (Figure 5A) and FI levels (Figure 5B), and therefore insulin resistance as determined by the HOMA-IR insulin resistance index (Figure 5C), the continuous TRF indeed afforded protection against these outcomes associated with obesogenic diets, similar to those seen in the LFD-AL group. Although the post-TRF group exhibited FBG levels comparable to the HFD-AL group, the FI and thus HOMA-IR were significantly improved compared to the HFD-AL group. These data indicate that TRF’s protective effect against the development of insulin resistance could persist, at least for 2 weeks following TRF withdrawal.

## 4. Discussion

Numerous studies suggest that continuous TRF is associated with improvements in the metabolic and immunologic parameters associated with obesity, but the post-effects after TRF cessation are largely unknown. In this study, we showed that, although TRF withdrawal quickly led to increased body weight and adiposity, and reversed FBG, mice in the post-TRF group exhibited FI and insulin resistance index HOMA-IR similar to the TRF group. While circulating monocytes and hepatic gene expressions regarding inflammation returned to the levels seen in the HFD-AL group, the post-TRF cohort exhibited adipose tissue inflammation and macrophage infiltration that was significantly less severe than in the HFD-AL group. These results suggest that (1) continuous TRF is most effective, (2) prior TRF effects last but vary depending on tissues, e.g., the resulting suppression on adipose tissue inflammation and insulin resistance could last a couple of weeks even with obesogenic diets, and (3) the cessation of TRF does not heighten the metabolic risk compared with sustained obesity.

Previous reports have shown that weight fluctuations due to alternating low-/high-fat diets worsens systemic glucose tolerance in rodents [22,23]. These studies also indicate that inflammatory phenotypes in adipose tissue were exacerbated in those with weight fluctuations compared with sustained obesity [23,24]. However, in our model, rather than drastic changes in diet composition, we employed a mouse model under the same obesogenic diet to define the role of TRF and its post-effects on the metabolic and immunologic parameters associated with obesity. We found that, although the post-TRF group had a slight fluctuation in body weight, their final weight was maintained at lower levels than those under chronic ad libitum feeding. In addition, the post-TRF group had insulin resistance index that was significantly less severe than the HFD-AL group, which had persistent weight gain. Moreover, this cohort exhibited a reduced gene expression of proinflammatory molecules *Adgre1* (F4/80) by 38.1%, *Itgax* (CD11c) by 57.0%, and *Tnf* by 30.5% in adipose tissue, compared to the HFD-AL group. One of the characteristic changes in adipose tissues during obesity is the accumulation of CD11c^+^F4/80^+^ macrophages, which express high levels of proinflammatory mediators [25]. In contrast, the conditional deletion of the CD11c gene in obese mice could reduce metabolic inflammation and normalize insulin sensitivity [26]. Previously, we demonstrated that TRF decreased the numbers of total and proinflammatory CD11c^+^ macrophages in adipose tissue, along with reduced *Adgre1* and *Itgax* transcripts [8]. Thus, the decreased expression of *Adgre1* and *Itgax* seen in the post-TRF group may contribute to a metabolically healthier phenotype (including insulin sensitivity), even after TRF cessation. We acknowledge that, preferably, the measurement of protein levels would have further verified these results, which is a limitation of the current study.

PPARγ is a transcription factor that belongs to the nuclear receptor superfamily. It is predominantly expressed in adipose tissue and, to a lesser extent, in the spleen and immune system [11]. PPARγ is required for adipocyte differentiation, the regulation of insulin sensitivity, lipogenesis, and adipocyte survival and function [27]. Previously, it was shown that PPARγ expression at both mRNA and protein levels was reduced in the adipose tissue of mice and humans with obesity [18]. These alterations appear to be associated with the dysfunction of adipose tissue, while the overexpression of *Pparg* in adipogenic precursors drives healthy adipose tissue expansion with fewer crown-like structures and reduced adipose tissue inflammation, as well as an improvement in glucose homeostasis [28]. In contrast, the loss of *Pparg* in adipogenic precursors triggers pathologic visceral expansion with HFD feeding [28]. Interestingly, the macrophage-specific depletion of *Pparg* in mice impaired the anti-inflammatory phenotype of adipose tissue macrophages, and predisposed them to the development of obesity and insulin resistance [29,30]. Although we cannot distinguish the effects on adipocytes or macrophages that are abundant in obese adipose tissue, the current results showed a significant decrease in *Pparg* mRNA levels in the adipose tissue of obese mice. Additionally, *Pparg* downregulation was restored to the control level completely by TRF and, to a lesser degree, after its cessation. Thus, resistance against *Pparg* downregulation may contribute to the sustained benefits associated with post-TRF.

Compared to PPARγ in adipose tissue, the basal expression of PPARγ is very low in the liver [31]. However, PPARγ is strongly upregulated in steatotic livers [19,32]. By using adenovirus or high-fat diet feeding, the overexpression of hepatic PPARγ on a PPARα^−/−^ background was shown to increase hepatic lipid accumulation and fatty liver phenotypes [33,34]. In contrast, mice lacking *Pparg*, specifically in the liver, were protected from hepatic steatosis [35]. In the current study, hepatic *Pparg* mRNA expression in the TRF group was as low as in the LFD-AL group, despite the obesogenic diet in the former. In addition, the post-TRF group had hepatic *Pparg* mRNA levels higher than the TRF group, but significantly lower than the HFD-AL group. These results indicate that post-TRF could maintain partial but significant protection against the upregulation of the hepatic *Pparg* mRNA level, thereby reducing the risk of hepatic steatosis.

PPARα is mainly present in metabolically active tissues including the liver, where it plays a pivotal role in fatty acid catabolism. Although the expression of PPARα in adipose tissue is much lower compared to PPARγ, there is evidence that PPARα activation may reduce adipose tissue inflammation during obesity by decreasing adipocyte hypertrophy, by regulating inflammatory gene expression via locally expressed PPARα, and/or by systemic events likely originating from the liver [19]. Conversely, adipose-specific PPARα^−/−^ mice had increased lipogenesis and a polarity shift toward inflammatory macrophages in adipose tissue [36]. Although we cannot completely rule out the possibility that post-TRF maintains its systemic effect through the liver PPARα, we observed no difference in *Ppara* mRNA levels in the liver between the HFD-AL and post-TRF groups. Instead, we found that the post-TRF group had *Ppara* mRNA levels in adipose tissue as high as those in the LFD-AL group, along with decreased inflammatory gene expression. Thus, TRF, even after cessation, could help maintain the *Ppara* level in adipose tissue, which possibly contributes to the reduced inflammatory status of the tissue.

Recently, it was shown that a fasting could influence the monocyte metabolic and inflammatory activity, and reduce the number of circulating monocytes, in lean mice and healthy humans [37]. However, genetically obese *ob/ob* mice, or mice with ad libitum feeding of HFD, had an enhanced production of monocytes in the bone marrow [38] which, in turn, potentiates macrophage accumulation in the adipose tissue [39,40]. We previously demonstrated that prolonged fasting on a daily basis by TRF could normalize monocyte production in bone marrow and thus, circulating monocytes in overweight and obese mice [10]. Here, we showed that the post-TRF group at 1 week after cessation had monocytes in circulation similar in quantity to the LFD-AL group as well as the TRF group. However, the post-effects did not last after 2 weeks of cessation, which indicates a catch up of macrophage accumulation in adipose tissues.

Despite the accumulating evidence of the metabolic benefits of TRF in rodent models, the effects of time-restricted eating in humans appear to be less obvious. Recent meta-analyses indicated that time-restricted eating resulted in a significant decrease in body weight and fat mass [41,42,43], but waist circumference, body mass index, fasting glucose, glycosylated hemoglobin, and blood pressures remained unchanged or had modest reduction depending on the study [41,42,43]. The differences in study design, including study duration, daily eating window, and compliance rates, among others, may in part account for the confusing results. For instance, time-restricted eating to the early part of the day was shown to improve fasting glucose and ameliorate inflammation markers in healthy subjects, but not in those under eating restricted to the middle of the day [44]. Thus, further clinical studies are needed to draw definitive conclusions for the usefulness of time-restricted eating in humans.

This study has several limitations. Firstly, our study included only male mice. Although female mice gained less weight than males upon HFD feeding [45], our results need to be confirmed as to whether this is also true in female models. Secondly, we did not include TRF and post-TRF regimens with LFD feeding. Since mice fed an LFD, even under ad libitum regimen, showed diurnal rhythms in food intake unlike those fed an HFD [2,4], we suspect that obese mice on TRF dramatically experience extended daily fasting and they benefit more against the metabolic inflammation associated with obesity. However, future studies employing an LFD regimen are necessary to confirm that TRF could deliver additional health benefits in the lean control. In addition, although circulating monocytes were measured for one and two weeks after the TRF cessation in obese mice, more extended time-courses of post-TRF were not performed in this study. Thus, the lasting effects on lean and obese mice could be different, which needs to be confirmed in a time-course of TRF cessation, for example, one, two, four weeks, and beyond. Finally, we measured metabolic and inflammatory markers in the blood after an 6 h fast (ZT23-ZT5) and in tissues harvested at ZT6. We chose an 6 h fast (morning fast) within a more physiological context since overnight fasting generates a starvation state in mice and could reduce lean body mass up to 15% [46,47]. It is noteworthy that, in mice fed an HFD ad libitum, the hepatic mRNA expression of *Pparg* was constitutively high, although rhythmic transcription existed [6]. Although we matched the fasting duration and time of day, it would be advisable to perform this at various time points, including during dark phases, to elaborate the effect of TRF and post-TRF in association with circadian rhythms. 

## 5. Conclusions

Our findings suggest that, while continuous TRF is the most effective, its health benefits, including the maintenance of insulin sensitivity, may last for a period of time after the cessation of TRF practice. Given the observed tendency for HFD-related indicators to rebound after TRF cessation, further studies are needed to determine how metabolic and immunologic parameters are changed during repeated TRF interventions and, furthermore, a longer follow-up period is necessary so that a better understanding for the effectiveness of TRF can be achieved.

## Data Availability

The data presented in this study are available on request from the corresponding author.

## Supplementary Materials

The following supporting information can be downloaded at: https://www.mdpi.com/article/10.3390/nu15112617/s1, Figure S1: Pearson correlation between circulating monocytes and (A) *Adgre1* and (B) *Itgax* mRNA expression in adipose tissue.
