# Supplementary material for: Post-Effects of Time-Restricted Feeding against Adipose Tissue Inflammation and Insulin Resistance in Obese Mice
Source: Nutrients. 2023 Jun 2;15(11):2617. doi: 10.3390/nu15112617 (PMC10255447; doi:10.3390/nu15112617)
Supplement: Supplementary file 1 [file nutrients-15-02617-s001.zip › nutrients-2400917-supplementary.pdf]

nutrients

Supplementary Materials

Post-effects of Time-restricted Feeding Against Adipose Tissue Inflammation and Insulin Resistance in Obese Mice

Narae Yun [1], Jiyeon Nah [1], Mi Nam Lee [2], Dayong Wu [3] and Munkyong Pae [1,*]

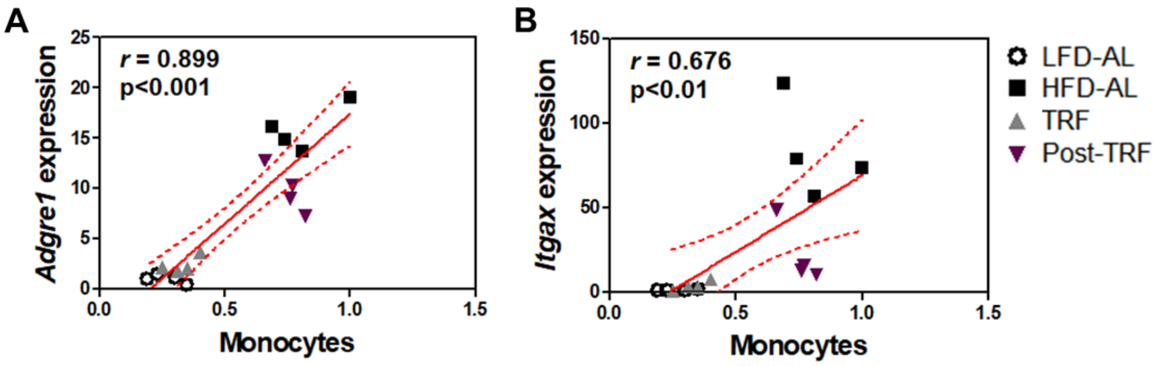

Figure S1. Pearson correlation between circulating monocytes and **(A)** *Adgre1* and **(B)** *Itgax* mRNA expression in adipose tissue on LFD-AL (open circles), HFD-AL (closed squares), TRF (gray triangles), or Post-TRF (purple reversed triangles), n= 4 per group. LFD-AL, low-fat diet ad libitum for 12 weeks; HFD-AL, high-fat diet ad libitum for 12 weeks; TRF, 6 weeks of time-restricted feeding in mice underwent ad libitum of high-fat diet for 6 weeks; Post-TRF, 4 weeks of TRF, followed by 2 weeks of ad libitum feeding in those underwent ad libitum of high-fat diet for 6 weeks.
